# Treatment of Atypical Ulnar Fractures Associated with Long-Term Bisphosphonate Therapy for Osteoporosis: Autogenous Bone Graft with Internal Fixation

**DOI:** 10.1155/2017/8602573

**Published:** 2017-11-29

**Authors:** Yohei Shimada, Tetsuhiro Ishikawa, Jun Endo, Jo Katsuragi, Toshiaki Kotani, Hitoshi Kiuchi, Kazuki Kuniyoshi, Seiji Ohtori

**Affiliations:** ^1^Orthopaedic Surgery, Sanmu Medical Center, Chiba, Japan; ^2^Orthopaedic Surgery, Seirei Sakura Citizen Hospital, 2-36-2 Eharadai, Sakura, Chiba 285-8765, Japan; ^3^Department of Orthopaedic Surgery, Graduate School of Medicine, Chiba University, 1-8-1 Inohana, Chuo-ku, Chiba 260-8670, Japan

## Abstract

Long-term bisphosphonate use has been suggested to result in decreased bone remodelling and an increased risk of atypical fractures. Fractures of this nature commonly occur in the femur, and relatively few reports exist to show that they occur in other bones. Among eight previous reports of atypical ulnar fractures associated with bisphosphonate use, one report described nonunion in a patient who was treated with cast immobilization and another described ulna nonunion in one of three patients, all of whom were treated surgically with a locking plate. The remaining two surgical patients achieved bone union uneventfully following resection of the osteosclerotic lesion and iliac bone grafting before rigid fixation. We hypothesized that the discontinuation of bisphosphonate therapy, the use of teriparatide treatment, and low-intensity pulsed ultrasound (LIPUS) might have been associated with fracture healing.

## 1. Introduction

Long-term bisphosphonate use has been suggested to result in suppressed bone remodelling and an increased risk of atypical fractures [1]. Fractures of this nature commonly occur in the femoral shaft or subtrochanteric region but rarely occur in the upper extremities [2]. We describe two patients who sustained atypical ulnar fractures that were successfully treated using autogenous bone grafts with internal fixation.

## 2. Case 1

**Figure 1:**
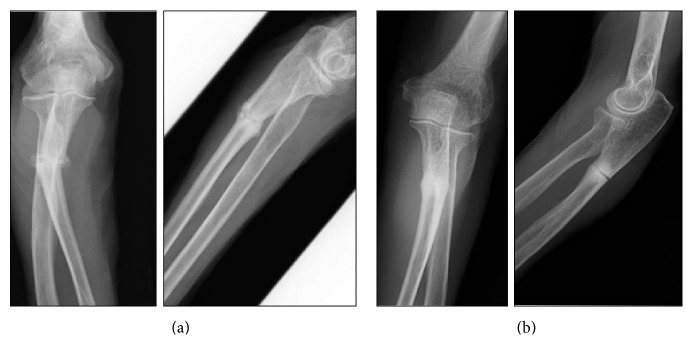
Plain radiographs of the right forearm ((a) case 1 and (b) case 2), showing a transverse fracture with cortical thickening in the proximal third of the ulna suggesting the presence of an atypical ulnar fracture.

**Figure 2:**
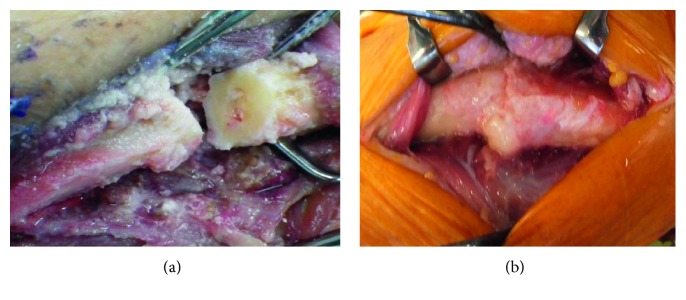
The pictures of the fracture site ((a) case 1 and (b) case 2). The area of the sclerotic bone was resected from the fracture site.

**Figure 3:**
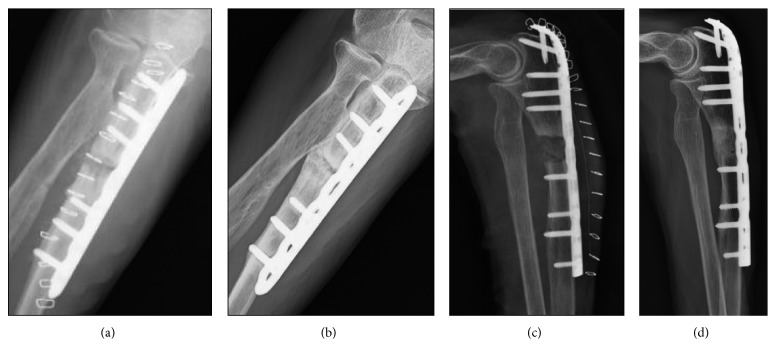
Plain radiographs of case 1 taken immediately after surgery (a) and 1 year after surgery (b) and case 2 taken immediately after surgery (c) and 1.5 years after surgery when bone union was observed gradually (d).

A 79-year-old woman with no history of trauma presented with pain in her right arm that had persisted for one month. Her medical case history revealed that, for the past 6 years, she had received 35 mg/week alendronate as treatment for osteoporosis. Plain radiographs showed a transverse fracture in the proximal third of her ulna, and cortical thickening was observed at the fracture site (Figure 1(a)). A baseline dual-energy X-ray absorptiometry (DXA) scan showed that her femoral neck bone mineral density (BMD) was 0.34 g/cm^2^. Blood tests indicated that her serum calcium, phosphate, and alkaline phosphatase levels were in the normal ranges. The patient was diagnosed with an atypical fracture of the ulna. The patient discontinued her alendronate regimen, and surgical treatment for her fracture was planned. Because the fracture site appeared similar to pseudarthrosis, a 5 mm area of the sclerotic bone was resected from the fracture site (Figure 2(a)), and an autologous corticocancellous iliac bone graft was inserted at the resection site and fixed with a locking plate (Figure 3(a)). She was given teriparatide daily for osteoporosis, and low-intensity pulsed ultrasound (LIPUS) was done after surgery. LIPUS was done once a day for 1 year, and bone union was observed 1 year after the surgery (Figure 3(b)).

## 3. Case 2

An 89-year-old woman complained of acute pain in her left arm after she lightly hit her elbow on a shelf. For the past nine years, she had been treated with 17.5 mg/week risedronate. Plain radiographs showed a transverse fracture in the proximal third of her ulna (Figure 1(b)). DXA revealed that her femoral neck BMD was 0.57 g/cm^2^, and blood tests showed normal results. The patient was diagnosed with an atypical fracture of the ulna. The risedronate was discontinued, and she was treated surgically. The sclerotic region was resected (Figure 2(b)), and an iliac bone graft was inserted followed by rigid fixation with a locking plate (Figure 3(c)). Daily teriparatide treatment was administered for the patient's osteoporosis, and an LIPUS was done once a day for one year. Bone union was observed 1.5 years after the surgery (Figure 3(d)).

## 4. Discussion

Bisphosphonates are one of the most widely prescribed drugs for the treatment of osteoporosis and the reduction of fracture risk. Prospective randomized clinical trials have been conducted to investigate bisphosphonate-related atypical fractures [3], although causality between the two has not been conclusively established. Atypical bisphosphonate-related fractures commonly occur in the femur. Fewer reports exist to show that atypical fractures occur in other bones, such as the clavicle, tibia, pelvis, radius, and ulna [4–6].

Eight reports of atypical ulnar fractures associated with bisphosphonate use have been published [2]. Ulnar fractures associated with bisphosphonate use are relatively rare and not well defined. The two cases in this report suggest that they display features similar to those described for atypical femoral fractures according to the revised case definition of atypical femoral fractures published in 2013 by the American Society for Bone and Mineral Research task force [7]. In particular, all fractures met the relevant major criteria: they were atraumatic, noncomminuted, and transverse in configuration and had localized periosteal or endosteal thickening of the cortex at the fracture site.

To date, there have been no guidelines published on the duration of therapy that may optimize the risk-benefit ratio and safety profile of bisphosphonate. In a systematic review of all case series and case reports of atypical femoral fractures, Park et al. reported a median time of 5 years of therapy prior to fractures [8]. The eight aforementioned reports of atypical ulnar fractures associated with bisphosphonate use were for durations of treatment far exceeding 7 years [2]. The two patients in this report received bisphosphonate for 6 years and 9 years, respectively, prior to ulnar fracture. Together, these cases suggest that atypical ulnar fractures may be a consequence of long-term bisphosphonate therapy.

Among the eight previously reported atypical ulnar fracture cases, nonunion occurred in one patient who was treated with cast immobilization, healing was incomplete in one of three patients surgically treated with a locking plate, and two patients were successfully treated by resecting the osteosclerotic lesion, inserting an iliac bone graft, and subsequent rigid fixation with a locking plate. Treatments for the remaining patients in those eight reports were not described [2, 9–11].

Some reports indicate that the discontinuation of bisphosphonate and the initiation of teriparatide treatment have been associated with fracture healing in atypical fracture cases; however, the majority of those reports lack sufficient evidence, few were randomized controlled trials, and many were case reports and case series [12–15]. Carvalho et al. reported that teriparatide had osteosynthetic effects on atypical femoral fractures associated with bisphosphonate use [13]. Nozaka et al. reported that teriparatide and LIPUS have contrasting, additive effects during fracture healing [14]. Teriparatide administration was used to treat osteoporosis in our report, rather than administered for fracture healing. Currently, the orthopaedic use of teriparatide, by physicians who are confident of its beneficial effects on the repair of fractures and nonunions when administered to patients, represents off-label use. Well-designed, randomized controlled trials are required to comprehensively analyze the actions of teriparatide in patients [15].

Atypical fractures can be treated conservatively or surgically based on the fracture site, fracture type, and the amount of displacement. The possible pitfalls of a surgical approach should be an important consideration when treating atypical fractures. However, in the eight previously discussed reports and the two cases featured in this report, unions were achieved without complications when the fractures were treated operatively with sequential resection of the osteosclerotic lesion, insertion of an iliac bone graft, and rigid fixation with a locking plate. Further investigations are needed to determine whether the combination of teriparatide and LIPUS is an effective treatment for atypical fractures.

In summary, this report suggests that atypical fractures associated with long-term bisphosphonate use can occur in bones other than the femur. Careful preoperative planning and modification of the operative technique may also be necessary.
